# Finding “truth” across different data sources

**DOI:** 10.1186/s13584-017-0138-3

**Published:** 2017-03-21

**Authors:** Alison Rein, Lisa A. Simpson

**Affiliations:** AcademyHealth, 1666K Street, Suite 1100, Washington, DC, 20006 USA

**Keywords:** Data Validation Study, Data Integration, Electronic Health Records, Patient Reported Outcomes, Cancer Survivorship

## Abstract

The proliferation of new technology platforms and tools is dramatically advancing our ability to capture, integrate and use clinical and other health related data for research and care. Another critical and increasingly common source of data comes directly from patients – often in the form of Patient Reported Outcomes (PRO). As more providers and payers recognize that patient experiences reflect a critical dimension of the value proposition, these data are informing broader strategies to achieve performance improvement and accountability in health systems. Combined with other traditional (e.g., claims) and more recent (e.g., Electronic Health Record) data assets, PROs can help to examine experiences and outcomes that convey a more complete picture of both individual and population health. One of the areas of research where this is most evident is cancer survivorship, including long-term adverse effects, as the population of survivors is increasing given advances in detection and treatment.

Key questions remain as to how and under what conditions these new data resources can be used for research, and which are the best “sources of truth” for specific types of information. A recent IJHPR validation study by Hamood et al. reflects important progress in this regard, and establishes the necessary groundwork for a larger planned study. There are some important limitations worth noting, such as a small sample size (which does not support adequate subgroup analysis); a relatively narrow focus on women with only early stage or regionally advanced breast cancer; and a limited focus on outcomes that are primarily clinical and relatively severe in nature (e.g., cardiovascular disease).

Finally, as use of EHRs becomes ubiquitous, as patient perspectives and outcome measures are considered, and as more types of data are systematically collected via electronic systems, further comparison and validation of non-clinical data elements captured via such tools will become increasingly possible and important. This will further enhance the capacity of cancer survivorship researchers to address a broader range of important questions to many more types of patients.

## Background

The proliferation of new technology platforms and tools is dramatically advancing our ability to capture, integrate and use clinical and other health related data for research and care. In the United States (US), the pace of technological innovation was accelerated by the policies and financial incentives offered to stimulate the adoption of electronic health records (EHR) established by the HITECH Act of 2009; it was further advanced by a number of provisions in the Affordable Care Act that leverage data systems to transition payments from volume to value.

Another critical source of data comes directly from patients – often in the form of Patient Reported Outcomes (PRO). These PRO data are of increasing interest, as more providers and payers recognize that patient experiences reflect a critical dimension of the value proposition. This is happening both in the US and globally, as part of broader strategies to achieve performance improvement and accountability in health systems [1–3]. Combined with other traditional (e.g., claims) and more recent (e.g., EHR) data assets, PROs can help to examine experiences and outcomes that convey a more complete picture of both individual and population health. One of the areas of research where this is most evident is cancer survivorship, including long-term adverse effects, as the population of survivors is increasing given advances in detection and treatment [4, 5]. A recent systematic evaluation of nearly 800 adverse events listed in the Common Terminology Criteria for Adverse Events (CTCAE) identified 78 appropriate for patient self-reporting [6]. Together, these policy shifts and technology trends are enabling unprecedented integrations of multiple data sources and systems to advance learning health systems for all patients, including those treated for cancer [7–9]. Key questions remain, however, as to how and under what conditions these new data resources can be used, and which are the best “sources of truth” for specific types of information.

## Discussion

We applaud the efforts of Hamood et. al. [10] to explore the validity of different data sources for use in cancer survivorship research; such assessments of data quality, completeness and comparability are critically important - both to understanding and characterizing existing data assets, and to further building a robust research data infrastructure. While that feasibility study, which was recently published in the Israel Journal of Health Policy Research, reflects important progress in this regard, some limitations are worth noting. For example, the study’s focus on women with only early stage or regionally advanced breast cancer limits the generalizability of findings, as women with more advanced disease may be particularly at risk of adverse events and poor outcomes and may be more or less willing to participate in PRO measurement. A related point is that, as a feasibility study, the sample size does not support sub-group analyses that would help identify patients less likely to participate in PRO studies or with different care experiences that could differ by age, cancer stage, or estrogen sensitivity. It may be that the data quality and completeness are similar for all regardless of such differences, but the lack of assessment in this work leaves unanswered questions – particularly for researchers wishing to conduct studies relevant to older and/or sicker patient populations using these data tools.

Also worth noting is that - to the extent that a primary aim of this study is to assess the comparability of administrative claims data relative to EHR data - the authors have a priori limited the outcomes of interest to those that are clinical and relatively severe in nature (e.g., cardiovascular disease). In this study, other important sequela (e.g., impact on relationships, employment) experienced by cancer survivors are captured via the self-reported questionnaire but, as indicated by the authors, such tools can only accommodate a small number of these questions without significantly increasing response burden. In neither case is it clear the extent to which patients and their caregivers were involved or consulted in the process of determining primary outcomes for assessment, but this is increasingly of interest – if not yet standard practice. Over time, as use of EHRs becomes ubiquitous, as patient perspectives and outcome measures are considered, and as more types of data are systematically collected via EHR systems, further comparison and validation of non-clinical data elements captured via such tools will become increasingly possible and important [11]. This will further enhance the capacity of cancer survivorship researchers to address a broader range of important questions to many more types of patients.

Finally, and perhaps most importantly, we wonder about the extent to which the methods applied and conclusions drawn from this effort will hold true when it is deployed across multiple institutions and with a far more diverse patient population. This is certainly an area of tremendous interest, and warrants further consideration.

## Conclusion

Leveraging multiple sources and types of data to assess and improve the quality and outcomes of care is now a fundamental strategy for any learning health system. As many of these sources are relatively new and rapidly evolving, efforts to understand underlying quality, reliability and feasibility of each data source is critical, as this small study demonstrates. Also worth noting is that this process of data source assessment and validation is likely to require continuous monitoring and updating; over time, and as health care providers are able to collect more and better quality data via EHRs (and more easily via natural language processing), the characteristics and applicability of data in these systems will evolve. The same holds true for data captured via personal devices and other novel sources that will enable researchers to more deeply explore the contexts and outcomes critical to patient health and wellbeing.

## Abbreviations

PRO: Patient Reported Outcomes
EHR: Electronic Health Record
CTCAE: Common Terminology Criteria for Adverse Events
